# The Association Between HIV/AIDS, Ototoxicity of Its Treatments, and Occupational Noise Induced Hearing Loss: A Narrative Review Mapping the Existing Literature and Identifying Research Gaps

**DOI:** 10.3390/ijerph22040487

**Published:** 2025-03-25

**Authors:** Katijah Khoza-Shangase

**Affiliations:** Audiology Department, School of Human & Community Development, Faculty of Humanities, University of the Witwatersrand, Johannesburg 2050, South Africa; katijah.khoza-shangase@wits.ac.za

**Keywords:** HIV/AIDS, occupational noise-induced hearing loss, antiretroviral therapy, ototoxicity, South Africa, hearing conservation, public health, sensorineural hearing loss

## Abstract

Hearing loss remains a significant but underexplored health challenge in individuals with HIV/AIDS, particularly those exposed to occupational noise. The ototoxic effects of antiretroviral therapy (ART) and comorbid conditions like tuberculosis (TB) further compound the risk. This narrative review examines the intersection of HIV/AIDS, ART, and occupational noise-induced hearing loss (ONIHL), emphasizing the South African and broader African contexts. The aim of the study was to map the existing literature on the association between HIV/AIDS, its treatments, and ONIHL, and to identify research gaps to inform policy and clinical practice. A narrative review approach was adopted, systematically searching databases including PubMed, Scopus, and Web of Science. Studies published between 2000 and 2024 were included, focusing on the effects of HIV/AIDS, ART, and occupational noise exposure on hearing health. Data extraction and thematic synthesis were performed to identify key findings and gaps. Twenty studies were included, covering diverse settings such as South Africa, Cameroon, Tanzania, and the USA. Three key themes emerged: (1) dual burden of HIV and occupational noise exposure: HIV-positive individuals in noise-intensive industries, such as mining, face amplified risks of hearing loss due to immunological compromise and ototoxic TB treatments; (2) ototoxicity of ART: older ART regimens, widely used in resource-limited settings, are associated with a higher prevalence of sensorineural hearing loss (SNHL); and (3) immunological susceptibility to ONIHL: HIV-related immune suppression exacerbates cochlear damage from noise and ototoxic agents, contributing to both peripheral and central auditory dysfunction. This review highlights the urgent need for integrated hearing health interventions in HIV care and occupational health frameworks, particularly in high-prevalence regions like South Africa. Routine audiological assessments, access to safer ART regimens, and enhanced workplace protections are essential to mitigate the dual burden of HIV/AIDS and ONIHL. Future research should prioritize longitudinal studies and innovative, low-cost solutions for resource-limited settings.

## 1. Introduction

Hearing loss is a significant global health challenge, with occupational noise-induced hearing loss (ONIHL) recognized as one of the most prevalent occupational health conditions worldwide [1,2,3,4,5], disproportionately affecting workers in industries such as mining, manufacturing, and construction. In sub-Saharan Africa, the burden of hearing loss is exacerbated by high rates of communicable diseases such as human immunodeficiency virus/acquired immunodeficiency syndrome (HIV/AIDS) and tuberculosis (TB) [6,7]. This is alongside the economic dependence on noise-intensive industries like mining [8,9,10]. The region’s unique convergence of occupational noise exposure, ototoxic drug use, and immunological compromise raises the need for a nuanced understanding of the intersection between these factors. In South Africa, this condition presents a significant public health burden, given the country’s reliance on noise-intensive industries such as mining [11,12]. Estimates indicate that ONIHL contributes significantly to disability-adjusted life years lost in the country, highlighting the need for robust hearing conservation programs (HCPs). Specifically, the complexity of the South African healthcare landscape, compounded by high rates of the listed communicable diseases, underscores the need for careful examination of how these comorbid conditions and their treatments interact with ONIHL. This review investigates the relationship between HIV/AIDS, its treatments, and ONIHL, with a particular focus on high-burden settings like South Africa, where these interactions present critical public health and occupational challenges.

HIV/AIDS continues to have a profound impact on public health in South Africa, home to the largest number of people living with HIV (PLHIV) globally [13]. South Africa is at the epicenter of the global HIV/AIDS pandemic, with an estimated 7.7 million individuals living with the disease. The introduction of highly active antiretroviral therapy (HAART) has transformed the prognosis for individuals with HIV/AIDS, reducing mortality and morbidity—from a fatal condition to a chronic, manageable illness [14]. However, ART is not without side effects. Evidence suggests that some regimens are associated with a range of ototoxic side effects, including auditory dysfunctions such as sensorineural hearing loss (SNHL) and tinnitus [15,16]. Several ART medications, particularly nucleoside reverse transcriptase inhibitors (NRTIs) and protease inhibitors (PIs), have been implicated in auditory dysfunction. Older-generation NRTIs such as zidovudine (AZT), stavudine (d4T), and didanosine (ddI) have been associated with SNHL, while certain protease inhibitors, including lopinavir/ritonavir and atazanavir, have shown potential ototoxic effects [16,17,18]. Additionally, non-nucleoside reverse transcriptase inhibitors (NNRTIs) such as efavirenz have been linked to central auditory processing deficits. Beyond ART, aminoglycoside antibiotics (e.g., amikacin, kanamycin, streptomycin) used to treat tuberculosis (TB)—a common comorbidity in HIV-positive individuals—are well-documented for their ototoxicity [7,11,12]. Studies like those by Fokouo et al. [17] and Khoza and Ross [18] have documented the intricate links between HIV infection, HAART, and auditory health, with evidence pointing to both direct viral impacts on the auditory system and the ototoxicity of certain antiretroviral drugs. This is particularly concerning in resource-constrained settings where older ART regimens remain widely used due to their cost-effectiveness [19]. Similarly, TB—a common comorbidity of HIV—is often treated with aminoglycosides, which are also highly ototoxic [20,21,22]. These dual burdens of disease and treatment amplify the risk of hearing loss in individuals with HIV/AIDS.

Moreover, the occupational landscape in South Africa presents unique risks for hearing health. Industries like mining, which are critical to the economy, expose workers to prolonged high-intensity noise levels [23,24]. For HIV-positive individuals employed in such sectors, noise exposure is compounded by immunological vulnerability and the ototoxic effects of ART and TB treatments [25]. This creates a perfect storm for auditory dysfunction, with workers facing heightened risks of both peripheral and central auditory pathway damage. While high-income countries (HICs) have implemented robust occupational safety regulations and advanced HCPs to mitigate such risks, these measures are often underdeveloped or inconsistently applied in low- and middle-income countries (LMICs) [26].

The interplay between HIV/AIDS, ART, and ONIHL is further complicated by immunosuppression caused by HIV [27,28]. The immune system’s role in maintaining auditory health has been well documented, with immune compromise linked to increased susceptibility to cochlear damage from both noise and ototoxic agents [27,29]. This is particularly relevant in the context of South Africa, where the prevalence of HIV/AIDS exceeds 7 million individuals, making immunosuppression a widespread concern [14]. The compounded effect of occupational noise exposure and HIV-related immunosuppression exacerbates cochlear vulnerability, contributing to long-term auditory impairments [25].

Despite the growing body of research on ONIHL and ototoxicity, the intersection of these conditions with HIV/AIDS remains underexplored, particularly in LMICs. The potential interaction between HIV/AIDS, its treatments, and occupational NIHL is underexplored in the global and South African contexts. Noise-induced cochlear damage, primarily affecting the outer hair cells, may be exacerbated by underlying conditions such as HIV-related immune suppression or mitochondrial dysfunction. Additionally, HAART, while effective in managing HIV, includes medications like tenofovir and nevirapine that have been implicated in auditory toxicity [30]. In South Africa’s mining sector—characterized by chronic noise exposure and a high prevalence of HIV/AIDS—these interactions could contribute to a cumulative burden of hearing loss among workers. Despite growing evidence on the auditory effects of HIV/AIDS and occupational noise exposure, significant gaps remain. For instance, the extent to which HIV-mediated immune changes exacerbate susceptibility to occupational noise remains unclear. Limited research has assessed the combined ototoxic effects of HAART and other noise-related exposures, such as mining equipment and blasting operations. The role of socioeconomic and occupational factors in amplifying these risks in South Africa’s vulnerable populations is poorly understood, and existing studies, such as those by Khoza-Shangase [12,28] and Brits et al. [11], highlight the need for a more integrated approach to hearing conservation that accounts for these complexities. This narrative review aims to address this gap by synthesizing existing evidence and identifying critical research gaps. Specifically, this review aims to map the existing literature on the association between HIV/AIDS, its treatments, and occupational noise-induced hearing loss; identify gaps in current research to inform policy and practice, with a focus on South Africa; and highlight the implications of these interactions for occupational health policies and HCPs in high-risk sectors.

By highlighting these intersections, this review seeks to inform policy and practice, advocating for routine audiological assessments, equitable access to safer ART regimens, and enhanced workplace protections. Furthermore, it emphasizes the importance of future research, particularly longitudinal studies and innovative, low-cost solutions tailored to resource-limited settings. In addressing these challenges, South Africa can lead global efforts in mitigating the dual burden of HIV/AIDS and ONIHL, improving the quality of life for affected individuals while advancing public health equity.

In a country grappling with dual epidemics of occupational NIHL and HIV/AIDS, the intersection of these conditions represents a critical public health challenge. By synthesizing existing knowledge and identifying gaps, this review seeks to pave the way for targeted research and intervention strategies to mitigate the burden of hearing loss in vulnerable populations. This work is particularly timely given South Africa’s policy shift toward universal healthcare and the implementation of the National Health Insurance (NHI) [31], which demands evidence-based, contextually relevant health interventions.

While previous studies have examined ONIHL, ART-related ototoxicity, and HIV-associated immunosuppression as separate contributors to auditory dysfunction, this review takes an integrated approach. The introduction is structured to first introduce the broader occupational health concern of ONIHL before discussing ART-related ototoxicity, which disproportionately affects HIV-positive individuals in occupational settings. Lastly, the role of HIV-related immunosuppression is considered as a compounding factor, further exacerbating susceptibility to hearing loss. This sequence reflects the multifaceted nature of the issue, ensuring that each contributing factor is contextualized within the broader framework of HIV, occupational health, and hearing conservation

## 2. Materials and Methods

### 2.1. Study Design

This study employed a narrative review approach to map the existing literature on the association between HIV/AIDS, its treatments, and ONIHL. A narrative review was chosen to provide a comprehensive synthesis of the available evidence, explore complex interrelationships, and identify gaps in knowledge. The method is particularly suited to this study’s objective of integrating research from diverse disciplines and sources, including audiology, occupational health, and infectious diseases. Narrative reviews are appropriate for such a task, providing a rich interpretation of the contemporary state of knowledge without the restrictions of strict rigid inclusion/exclusion criteria typically found in systematic reviews [32].

### 2.2. Literature Search Strategy

The literature search for this narrative review was iterative and comprised multiple cycles of searching, analysis, and interpretation. Following recommendations by Levac, Colquhoun, and O’Brien [33], while ensuring feasibility, the search strategy was designed to be clear, transparent, as well as comprehensive. The literature search was conducted in December 2024 across several electronic databases. The process involved the following:

- Databases Searched: Literature was sourced from multiple academic databases, including ScienceDirect, CINAHL, EBSCOHost, MEDLINE, ProQuest, PubMed, Scopus, Google Scholar, and African Journals Online (AJOL).
- Keywords and Boolean Operators: Search terms were developed using a combination of keywords and Medical Subject Headings (MeSH) terms, including the following:
  ○ HIV/AIDS (“HIV infection”, “HIV treatment”, “antiretroviral therapy”, “HAART”);
  ○ Occupational Noise-Induced Hearing Loss (“NIHL”, “noise exposure”, “occupational hearing loss”);
  ○ Combined Terms (“HIV and hearing loss”, “ototoxicity and noise”, “HIV treatment and noise-induced hearing loss”) with Boolean operators (AND, OR) were used to link search terms for inclusivity.
- Time Frame: Articles published between 2000 and 2024 were considered to ensure the inclusion of both historical and recent evidence.

### 2.3. Study Selection

This study adhered to the following overall inclusion and exclusion criteria:

Inclusion Criteria:

- Publications focusing on the auditory effects of HIV/AIDS, its treatments, and occupational noise exposure.
- Research conducted in human populations, including clinical, occupational, and community-based studies.
- Publications in English, with a particular emphasis on studies from South Africa and other sub-Saharan African countries.
- Peer-reviewed articles, gray literature, and reports from credible organizations.

Exclusion Criteria:

- Studies focusing solely on ototoxicity without mentioning occupational noise exposure anywhere.
- Animal studies or in vitro research.

This narrative review was conducted as part of a collaborative research cluster, with two additional research team members involved in the literature search, selection, and review process. The team worked collectively to ensure a comprehensive and balanced selection of studies, minimizing the risk of bias and omission. Each identified study was reviewed by at least two members, with discussions held to reach consensus on inclusion and exclusion decisions. This approach ensured the integrity and rigor of the literature selection, aligning with best practices in narrative reviews.

The initial literature search yielded 151 publications. Given the iterative nature of narrative reviews, additional searching was halted once saturation was reached—when no new themes or insights emerged from the included literature. At this point, 131 publications were excluded for the following reasons:

- They did not specifically examine the intersection of HIV/AIDS, its treatments, and ONIHL.
- They focused solely on either HIV/AIDS-related ototoxicity or ONIHL without considering their combined effects.
- They lacked relevance to the South African or broader African context, where the burden of both HIV/AIDS and ONIHL is particularly pronounced.

Ultimately, 20 studies were included in the review, providing a sufficient and comprehensive synthesis of the key themes and insights relevant to understanding the relationship between HIV/AIDS, HAART, and ONIHL, particularly in high-prevalence and resource-limited settings like South Africa.

### 2.4. Data Extraction and Synthesis

Guided by Peters et al.’s [34] guidelines, data were mapped in accordance with this review’s objectives, though the use of a standardized data extraction form. Full-text articles were assessed for eligibility based on the inclusion and exclusion criteria, and a total of 20 studies formed the final evidence data used in the review and are summarized and presented in Table 1. The review process involved extracting key information from each identified source, including the following:

- Researcher(s) and year of publication;
- Title of the study;
- Country and setting of the study;
- Population characteristics (e.g., age, gender, occupation);
- Study objectives;
- Key findings (specific to HIV/AIDS, treatments, and ONIHL);
- Conclusions and recommendations.

### 2.5. Data Analysis

A narrative approach was adopted to analyze the extracted data, enabling an in-depth interpretation of findings [49,50]. The analysis involved the following steps:

- Thematic Organization, where extracted data were categorized into recurring themes to synthesize the current knowledge base. These themes included the impact of HIV/AIDS on auditory health; the ototoxicity of ART; and interactions between occupational noise exposure and HIV-related hearing loss.
- Saturation, where saturation was achieved when additional studies no longer introduced new insights or themes, ensuring thematic sufficiency [32]. Emerging themes, such as the dual burden of noise and ototoxicity, consistently appeared across multiple studies, signaling that the literature adequately addressed the research question.
- Iterative Refinement, where the search and analysis process were iterative, with new studies integrated until no substantial additional information was identified.

### 2.6. Quality and Rigor

Although narrative reviews are inherently more flexible than systematic reviews, steps were taken to ensure quality and reliability [51]. Firstly, transparent documentation was carried out where the search strategy, study selection criteria, and data extraction process were thoroughly documented for reproducibility. Secondly, critical appraisal occurred, where each included study was assessed for the relevance and applicability of their findings to the current research question. Lastly, iterative refinement occurred, where the research question and scope were continuously refined as the literature review progressed, ensuring alignment with emerging evidence and themes. To ensure rigor in the review process, literature selection was carried out collaboratively within a research cluster. At least two team members independently reviewed each study, with discrepancies resolved through discussion and consensus. This iterative approach enhanced the reliability of findings and reduced the likelihood of bias. Although a formal systematic review protocol was not followed, the adherence to transparent selection and evaluation methods ensured a structured and robust narrative review.

### 2.7. Reflexivity and Interpretation

Reflexivity was maintained throughout the review to account for the researcher’s perspective, contextual influences, and potential biases. This involved (1) contextual consideration where the researcher recognized the unique challenges of South African healthcare systems, including the high prevalence of HIV/AIDS and ONIHL in sectors like mining, and (2) multiple perspectives where findings from diverse disciplines and sources were synthesized to provide a balanced interpretation of the literature.

### 2.8. Ethical Considerations

While ethical approval was not required for this study since it involved a review of the existing literature, ethical research practices were strictly followed [52,53,54], and these included (1) integrity and transparency where all sources were properly cited, and findings were presented objectively to ensure accuracy and credibility; (2) disclosure where potential conflicts of interest were disclosed to maintain transparency; and (3) academic rigor where the review process adhered to principles of academic rigor, including systematic data collection, objective synthesis, and ethical presentation of findings.

## 3. Results and Discussion

This narrative review included 20 studies from peer-reviewed journal articles, systematic reviews, and the relevant gray literature, examining the relationships among HIV/AIDS, ART, ONIHL, and ototoxicity. Studies were conducted across diverse settings, including Africa (South Africa, Cameroon, Tanzania, and Namibia), the Americas (USA and Brazil), and Europe (Germany), reflecting a diverse range of socioeconomic and clinical settings. The studies ranged from clinical and occupational health investigations to broader reviews of ototoxicity and auditory impairments among PLHIV.

Analysis of the characteristics of included studies revealed that, firstly, as far as geographic distribution was concerned, South Africa accounted for the majority of studies (8/20), emphasizing the country’s unique high burden of HIV/AIDS and its reliance on noise-intensive industries like mining. Other sub-Saharan African countries (e.g., Cameroon, Tanzania, Namibia) were also represented and provided additional insights into regional challenges, particularly in resource-limited settings, alongside studies from HICs (e.g., the USA, Germany). Studies from the USA and Europe offered comparative perspectives on the interplay between HIV, treatments, and hearing health in HIC settings. Secondly, when it comes to study populations, most studies focused on adults living with HIV/AIDS, often stratified by treatment status (e.g., HAART vs. no HAART). Occupational groups, such as miners and industrial workers, were frequently represented due to their high exposure to noise and increased prevalence of HIV/AIDS. Key demographic characteristics varied widely, with most studies involving adult populations and some examining mixed-gender cohorts. Lastly, when it comes to *methodologies*, a mix of cross-sectional, cohort, and case–control studies was included. Audiometric evaluations, patient interviews, and secondary data analyses were commonly employed. These studies offered diverse insights into the complex relationships among HIV/AIDS, ART, and ONIHL, as illustrated by evidence presented in Table 1.

The thematic analysis of the data in Table 1 and the publications summaries identified three key themes that address the research question: “What evidence exists on the relationship between HIV/AIDS, its treatments, and ONIHL, particularly in the context of South Africa and other resource-limited settings?”: (1) the dual burden of HIV/AIDS and occupational noise exposure on hearing health, (2) ototoxicity of ART, and (3) interactions between HIV-related immunological changes and noise-induced hearing loss.

**Theme** **1.** *The Dual Burden of HIV/AIDS and Occupational Noise Exposure on Hearing Health.*

Studies highlighted the amplified risk of hearing loss in individuals exposed to both occupational noise and HIV/AIDS. For example, multiple studies (e.g., [11,12,34]) demonstrated that workers in noise-intensive industries with HIV/AIDS were at a significantly higher risk of hearing loss. Khoza-Shangase [12] identified compounded risks of NIHL in South African miners with HIV/AIDS, emphasizing the synergistic effects of chronic noise exposure and immunological compromise. Brits et al. [11] and Khoza-Shangase [12] found that gold miners with TB (receiving aminoglycosides for TB treatment), a common comorbidity of HIV, exhibited significantly worse hearing thresholds compared to their counterparts without TB because of the compounding impact of ototoxicity. In South Africa, structural inequities in the mining sector exacerbate the vulnerability of HIV-positive workers to ONIHL. These inequities manifest in limited enforcement of occupational health policies, inconsistent provision of protective equipment, and socioeconomic barriers to accessing routine health monitoring. Addressing these disparities requires targeted interventions that prioritize the needs of this high-risk population, including enforcing existing noise regulations and expanding workplace-based hearing health programs.

The dual burden of HIV/AIDS and occupational noise exposure is particularly acute in South Africa due to the confluence of a high prevalence of HIV/AIDS and reliance on mining, a noise-intensive industry. This burden is particularly concerning in South Africa, where mining remains a cornerstone of the economy, employing large numbers of HIV-positive workers [23,24]. In South Africa, mining and similar industries are vital to the economy but pose substantial health risks. Workers with HIV/AIDS face dual vulnerabilities, chronic noise exposure and systemic immunosuppression, both of which exacerbate the risk of sensorineural hearing loss. Mining workers often experience prolonged exposure to noise levels exceeding occupational safety limits, which independently poses a significant risk for SNHL [35]. When combined with the immunological compromise associated with HIV/AIDS, the cochlea becomes even more susceptible to damage. This challenge is further compounded by TB, a common comorbidity, which requires ototoxic treatments like aminoglycosides. Globally, similar patterns have been observed in noise-intensive industries, though the scale of the problem varies and globally, low-resource settings share similar burdens. In contrast, HICs like the United States and Germany have successfully implemented stringent occupational safety regulations and advanced HCPs [36]. These interventions have significantly mitigated ONIHL risks, offering a model that South Africa and other LMICs can adapt. While resource constraints present challenges, localized adaptations of these strategies could help bridge the gap in occupational health outcomes. By contrast, South African miners often lack consistent access to protective equipment and regular hearing assessments, compounding the issue [37]. The implications are profound: without integrating auditory health into broader HIV care and occupational health frameworks, workers in high-risk industries face compounded vulnerabilities. This highlights the urgent need for policies that address the intersection of occupational health and chronic disease management. South Africa’s high prevalence of HIV/TB and reliance on mining demands urgent policy reforms to integrate hearing conservation into occupational and public health systems.

In South Africa, the mining industry accounts for a significant proportion of occupational illnesses, with ONIHL being among the most common [12]. The country’s NHI initiative provides an opportunity to integrate HCPs into broader occupational and HIV/AIDS health frameworks. Locally developed interventions, such as mobile health (mHealth) solutions and drive-through hearing clinics [55], could address barriers like geographic inaccessibility and lack of audiological expertise in rural areas. These findings have global implications. South Africa’s high prevalence of HIV/AIDS and its economic reliance on mining offer a unique lens through which to study the compounded risks of ONIHL and HIV-related auditory damage. Lessons learned from this context could inform global best practices, particularly in other LMICs with similar challenges. Community-based interventions, including educational campaigns for miners and employers, could play a pivotal role in mitigating ONIHL. These campaigns should focus on raising awareness of ONIHL risks, the importance of hearing protection, and the availability of audiological services. By involving community leaders and industry stakeholders, such initiatives could ensure greater uptake and sustainability.

Studies conducted in South Africa have emphasized the disproportionate burden of hearing loss among workers in noise-intensive industries, particularly mining. The intersection of high HIV prevalence and occupational noise exposure places miners at heightened risk, with studies such as Khoza-Shangase [28] and Brits et al. [11] demonstrating that HIV-positive workers are more susceptible to ONIHL. Despite the country’s comprehensive ART rollout, many workers remain on older, ototoxic regimens due to economic constraints. This highlights the urgent need for policies that integrate occupational noise management with routine HIV care. Beyond South Africa, studies in Cameroon, Tanzania, and Namibia have focused primarily on the ototoxic effects of ART and TB treatments rather than occupational noise exposure. Fokouo et al. [17] found that 63% of HIV-positive individuals in Cameroon exhibited SNHL, with similar findings in Tanzanian cohorts [38]. These studies highlight the importance of including hearing assessments as part of HIV care across Africa, even in non-noise-intensive occupational settings. Studies from other LMICs, such as Brazil, have examined hearing loss in HIV-positive populations, but with little focus on occupational noise exposure. These findings highlight a broader gap in research across LMICs, where the interaction between HIV, ART, and ONIHL remains underexplored. Limited access to audiological assessments and safer ART regimens further complicates the management of hearing health in these settings.

**Theme** **2.** *Ototoxicity of antiretroviral therapy (ART).*

Multiple studies such as Fokouo et al. [17], Maro et al. [38], and Torre et al. [39] documented significant associations between ART and auditory dysfunction, with older ART regimens like NRTIs and PIs showing higher ototoxicity rates. Key findings include that Fokouo et al. [17] reported that 63% of HIV-positive individuals on HAART in Cameroon exhibited hearing impairments, predominantly SNHL; that Torre et al. [39] demonstrated a higher prevalence of low-frequency SNHL in HIV-positive individuals on ART compared to HIV-negative controls; and that Maro et al. [38] highlighted that 63% of HIV-positive individuals on ART in Tanzania exhibited SNHL, predominantly linked to ototoxic ART and disease progression.

ART has revolutionized HIV care globally and has been a cornerstone of HIV management transforming the disease from a terminal illness to a manageable chronic condition but not without side effects. This progress has come at a cost, with older ART regimens—commonly used in LMICs due to their affordability—being associated with significant ototoxicity [56]. These findings are consistent with studies by Schouten et al. [57], which demonstrated auditory impairments in patients on zidovudine and didanosine. ART-related ototoxicity poses unique challenges in resource-limited settings like South Africa. While newer ART regimens have lower ototoxic risks, many public health programs in sub-Saharan Africa still rely on older, more cost-effective treatments with higher toxicity profiles. This highlights a critical disparity in healthcare quality between HICs and LMICs. This inequity raises the urgent need for policy reform to ensure the equitable distribution of safer ART regimens globally. While older ART regimens have been lifesaving in resource-constrained settings, their associated ototoxic risks present a significant public health concern. Transitioning to newer, less ototoxic ART regimens must be carefully evaluated, considering both the economic constraints of LMICs and the long-term health benefits of reduced auditory complications. Risk–benefit assessments should guide national HIV treatment policies, ensuring that the shift to safer ART regimens does not compromise treatment accessibility.

Globally, in HICs, advancements in ART formulations and access to less ototoxic ART regimens (e.g., integrase inhibitors), have reduced the prevalence of ototoxic side effects, but equitable access to these newer treatments remains a challenge. Locally, South Africa must prioritize the rollout of safer ART regimens and incorporate regular audiometric monitoring into HIV care protocols. For the rest of the LMICs, scaling up access to safer ART regimens, alongside routine audiological monitoring, is also crucial to minimizing hearing loss risks.

South Africa’s ART program is one of the largest globally, serving over 5 million people [14]. However, routine audiological monitoring is not a standard component of HIV care, leaving many at risk for undiagnosed hearing loss [28]. Programs like the National Consolidated Guidelines for the Management of HIV in Adults, Adolescents, Children and Infants and Prevention of Mother-to-Child Transmission could address this gap by incorporating hearing assessments into existing HIV care protocols, what Sebothoma [40] and Sebothoma and Khoza-Shangase [58] call a programmatic approach to ear and hearing care. The global implications are that the current findings reinforce the need for global collaborations to prioritize research into the ototoxic effects of ART, particularly in LMICs. Advanced data analytics and predictive algorithms could also be leveraged to identify patients at higher risk for ART-induced ototoxicity, enabling targeted interventions. An emerging area of innovation is pharmacogenetic screening, which could help identify individuals at higher risk of ART-induced ototoxicity. By tailoring treatment plans to genetic profiles, healthcare providers can minimize auditory side effects while maintaining ART efficacy. Although currently limited to high-resource settings, advancing the accessibility of such technologies could revolutionize HIV care in LMICs.

**Theme** **3.** *Interactions between HIV-related immunological changes and noise-induced hearing loss.*

HIV-induced immunosuppression was found to exacerbate the impact of noise on auditory health, emphasizing the role of HIV-related immunosuppression in exacerbating susceptibility to noise-induced hearing loss. The immunological compromise associated with HIV disrupts normal cochlear function, increasing vulnerability to both noise and ototoxic agents. The interplay between HIV-related immunological compromise and cochlear vulnerability involves complex mechanisms, including mitochondrial dysfunction and disrupted cellular repair pathways. These factors leave the auditory system more susceptible to noise-induced damage, highlighting the need for further research into the biological underpinnings of this heightened vulnerability.

Notable findings include those by De Jong et al. [30], who found that HIV-related immunological changes affect both peripheral and central auditory pathways, increasing susceptibility to noise-induced damage. Khoza-Shangase [41] and Brits et al. [11] emphasized the role of immune compromise in amplifying cochlear damage from noise exposure. These authors emphasized the vulnerability of immunocompromised individuals to auditory damage, particularly when exposed to high-intensity occupational noise. These findings align with earlier studies, such as Marra et al. [59], which found evidence of auditory processing deficits in HIV-positive individuals. Immunosuppression impairs the cochlea’s natural repair mechanisms, leaving it vulnerable to sustained damage from occupational noise and ototoxic drugs.

HIV-induced immunosuppression directly impacts auditory health by increasing vulnerability to both ototoxic agents and noise exposure [42,43,44,45,46,47,48,60]. In South Africa, where HIV prevalence is among the highest globally, this interaction poses a significant challenge, especially for workers in high-noise industries, and also represents a significant public health challenge. Immunological factors may amplify cochlear susceptibility to noise-induced damage, necessitating tailored interventions. Globally, this phenomenon is less studied in low HIV-prevalence settings, highlighting a critical gap in research and highlighting the need for targeted research. South Africa’s unique epidemiological context offers an opportunity to lead global efforts in understanding and addressing these complex interactions. While South Africa’s mining sector amplifies the risks of ONIHL, other African countries with high HIV prevalence but lower occupational noise exposure face unique challenges. For instance, agricultural or informal sector workers may encounter different environmental risks that interact with HIV-related immunosuppression. Comparative studies across these contexts could provide valuable insights into tailored prevention strategies.

Findings from this narrative review raise several implications. Locally, (1) policy integration with integration of HCPs into existing occupational health and HIV/AIDS management frameworks; (2) workplace protections with advocacy for stricter occupational noise regulations and enforcement of HCPs in industries with high HIV prevalence with safer ART regimens; (3) healthcare interventions with enhanced training for audiologists and occupational health practitioners to address the dual burden of ONIHL and HIV/AIDS including protocols for identifying and managing ART-and noise-related hearing loss, as well as routine audiological assessments being a mandatory component of HIV care, particularly for patients on ototoxic ART or TB treatments; and (4) innovative, cost-effective technologies, such as tele-audiology, should be scaled up to address geographic and systemic barriers. Globally, (1) research priorities with the need for international collaborations to study and address the compounded effects of HIV/AIDS and ONIHL as well as a focus on the development of predictive tools for hearing loss risk management; (2) equitable access with promotion of equitable access to newer safer ART regimens across LMICs to reduce ototoxicity-related disparities; (3) collaborative frameworks with multinational collaborations facilitating the development of standardized protocols for monitoring auditory health in HIV-positive individuals as well as standardized protocols for hearing conservation in HIV care; and (4) global standards for HCPs in HIV care should be developed, informed by experiences from high-burden regions like South Africa.

This narrative review has several limitations that must be taken into consideration. First, while efforts were made to include diverse studies, the reliance on English-language publications may have excluded relevant research from non-Anglophone regions. Second, the heterogeneity of methodologies across the included publications limited direct comparisons and synthesis of findings. Third, many studies focused on specific populations, such as miners or clinical cohorts, which may not represent the broader HIV-positive population. Finally, the absence of longitudinal studies in this field restricts the ability to establish causal relationships between HIV/AIDS, ART, and ONIHL. Despite these limitations, this review provides valuable insights and highlights critical research gaps for future exploration.

In high-income countries, studies have primarily focused on the clinical effects of HIV/AIDS on hearing rather than occupational noise exposure. For example, Torre et al. [46] and de Jong et al. [30] demonstrated an increased prevalence of SNHL in HIV-positive individuals but did not account for the effects of workplace noise exposure. These studies benefit from access to newer ART regimens with reduced ototoxicity and well-developed hearing conservation programs, presenting a stark contrast to the challenges faced in LMICs.

## 4. Conclusions and Recommendations

This review uniquely bridges the gap between clinical and occupational health perspectives, offering a comprehensive synthesis of the intersection of HIV/AIDS, ART, and ONIHL. By integrating evidence from diverse settings and populations, it provides actionable insights for addressing a significant yet underexplored public health challenge. This narrative review explored the complex interplay among HIV/AIDS, its treatments, and ONIHL, with a focus on understanding these relationships within the South African and broader African contexts. The findings highlight the compounded risks of hearing loss in HIV-positive individuals due to the synergistic effects of occupational noise exposure, ototoxic ART, and immunosuppression. The review also highlights the significant disparities in hearing health care access and resources between HICs and LMICs, further exacerbating the burden of auditory impairments in vulnerable populations.

The findings of this review highlight significant disparities in the study of HIV/AIDS-related hearing loss across different geographical contexts. In South Africa, where occupational noise exposure and HIV prevalence are both high, urgent policy interventions are needed to integrate hearing conservation into routine HIV care. In other African countries and LMICs, most research has focused on the ototoxic effects of ART and TB treatments, with limited attention given to workplace noise exposure. This gap highlights the need for studies that expand beyond clinical settings to consider occupational risks. By contrast, research in high-income countries has primarily examined hearing loss in HIV-positive populations without addressing the role of occupational noise. These differences emphasize the necessity of region-specific approaches to addressing the intersection of HIV/AIDS, ART, and ONIHL, ensuring that hearing health policies are aligned with local risk factors and healthcare capacities.

Key conclusions in as far as the dual burden of HIV and noise exposure firstly show that the convergence of high HIV prevalence and noise-intensive industries like mining in South Africa amplifies the risk of hearing loss. Immunological compromise and ototoxic treatments exacerbate cochlear vulnerability, creating a unique occupational health challenge. Secondly, as far as ototoxicity of ART and TB treatments is concerned, older ART regimens and TB medications, such as aminoglycosides, remain widely used in resource-constrained settings despite their ototoxicity. This raises the need for widespread adoption of safer drug alternatives. Lastly, when it comes to gaps in monitoring and prevention, routine audiological assessments are often absent in HIV care protocols, leaving many patients at risk for undiagnosed hearing impairments. Integrating hearing conservation strategies into HIV and occupational health frameworks is imperative.

Thus, recommendations include (1) policy and framework integration where policies that integrate hearing health into HIV care, occupational safety, and public health systems are developed and implemented, and where workplace HCPs are expanded to include regular audiological assessments for HIV-positive workers; (2) equitable access to safer treatments where widespread availability of newer, fewer ototoxic ART regimens in LMICs are advocated for, and where monitoring protocols for ototoxicity in patients undergoing TB treatment are incorporated; (3) education and training where healthcare providers and occupational health practitioners are trained to recognize and manage the auditory impacts of HIV/AIDS, ART, and noise exposure, and where patients are educated about the risks of hearing loss and the importance of regular hearing assessments; (4) leveraging technology where tele-audiology and mHealth platforms are used to improve access to hearing health services, especially in rural and underserved areas, and where predictive analytics tools are developed to identify individuals at high risk for hearing loss due to combined HIV, ART, and noise exposure; and (5) future research directions where longitudinal studies are conducted to establish causal relationships between HIV/AIDS, its treatments, and ONIHL; where the combined impacts of ART, noise exposure, and comorbidities like TB in diverse populations are investigated; and where innovative, low-cost interventions tailored to resource-limited settings are explored. When it comes to future research prioritizing longitudinal studies to track auditory health outcomes in HIV-positive populations exposed to occupational noise, collaborations with local mining companies and public health institutions could provide the infrastructure for robust data collection and intervention development tailored to the South African context.

The intersection of HIV/AIDS, ART, and ONIHL represents a critical public health challenge that demands urgent attention. By addressing these issues through integrated policies, equitable treatment access, and targeted interventions, South Africa can lead global efforts to mitigate the burden of hearing loss in HIV-positive populations. The findings of this review have direct implications for South Africa’s NHI initiative, which seeks to expand access to equitable healthcare. Incorporating HCPs and safer ART regimens into the NHI framework could significantly reduce the burden of ONIHL and improve overall quality of life for HIV-positive individuals. These efforts will not only improve the quality of life for affected individuals but also contribute to broader health equity and sustainability goals.

## Figures and Tables

**Table 1:** Summary of evidence related to HIV/AIDS, its treatments, and their interplay with occupational NIHL.

Researcher(s) and Year	Title of Study	Country and Setting	Population Characteristics	Study Objectives	Key Findings	Conclusions and Recommendations
Brits et al. (2012) [11]	Hearing profile of gold miners with and without tuberculosis	South Africa; mining settings	Gold miners with and without TB	To compare hearing profiles in miners with and without TB	Miners with TB had worse hearing thresholds due to ototoxic TB treatments	Calls for integrated auditory care in occupational health policies
Fokouo et al. (2015) [17]	Effect of HIV infection and highly active antiretroviral therapy on hearing function	Cameroon; clinical setting	HIV-positive adults on HAART; control group	To evaluate hearing function in HIV-positive individuals	63% of HIV+ individuals exhibited hearing impairments, predominantly SNHL	Highlights the need for hearing assessments in HIV management
Kho-za-Shangase et al. (2020) [26]	Occupational hearing loss in Africa: An interdisciplinary view of the current status	Africa; review study	Workers in various noise-exposed occupations	To review occupational hearing loss trends and contributing factors in Africa	NIHL remains under-reported; comorbid conditions like HIV/TB exacerbate risks	Urges better data collection and policy integration for hearing conservation
Khoza-Shangase (2020) [28]	Burden of disease: A scoping review of HIV/AIDS and TB in occupational noise-induced hearing loss	South Africa; healthcare and mining settings	Workers exposed to occupational noise; HIV/TB patients	To explore the intersection of HIV/AIDS, TB, and NIHL	HIV/AIDS and TB treatments increase susceptibility to NIHL due to ototoxicity and immune compromise	Urges the integration of hearing conservation into occupational and HIV care frameworks
de Jong et al. (2019) [30]	Main aspects of peripheral and central hearing system involvement in unexplained HIV-related hearing complaints	Germany; clinical setting	HIV+ adults with unexplained hearing complaints	To investigate the impact of HIV on central and peripheral auditory pathways	Both peripheral and central auditory impairments were noted	Suggests in-depth studies on HIV-related auditory dysfunctions
Khoza-Shangase & Moroe (2022) [34]	Occupational noise-induced hearing loss: An African perspective	South Africa; occupational health focus	Workers in mining and industrial sectors	To explore the prevalence and contributing factors of ONIHL in Africa	ONIHL risks are amplified in workers with comorbid HIV/TB	Advocates context-specific HCPs
Clark (2004) [35]	Otoacoustic emission testing in the early identification of noise-induced hearing loss in South African mineworkers	South Africa; mining settings	Mineworkers exposed to noise	To explore the use of otoacoustic emissions (OAEs) for early NIHL detection	OAEs detected early-stage hearing loss before symptoms became apparent	Suggests incorporating OAE testing in HCPs for miners
Wallhagen (2020) [36]	Hearing impairment and noise pollution: A behavioral medicine perspective	USA; behavioral study	General population exposed to noise pollution	To examine the impacts of noise pollution on health, including hearing loss	Chronic noise exposure can worsen auditory health in vulnerable populations	Calls for stricter noise regulations to protect public health
Hermanus (2007) [37]	Occupational health and safety in mining: Status, new developments, and concerns	South Africa; mining industry	Miners exposed to occupational hazards	To discuss occupational health and safety in the mining sector	Mining environments pose significant risks for NIHL, especially with HIV prevalence	Recommends integrating NIHL prevention into broader occupational health frameworks
Maro et al. (2014) [38]	Auditory impairments in HIV-infected individuals in Tanzania	Tanzania; hospital setting	HIV+ adults	To assess the prevalence of auditory impairments in HIV-infected individuals	High prevalence of SNHL linked to HIV progression and ototoxic ART	Recommends early and continuous auditory monitoring for HIV+ patients
Torre et al. (2015) [39]	Hearing loss among HIV-seropositive and HIV-seronegative men and women	USA; clinical and community settings	Mixed-gender cohort; HIV-positive and -negative adults	To assess differences in hearing loss prevalence between HIV+ and HIV- groups	HIV+ individuals demonstrated higher rates of SNHL, particularly at low frequencies	Recommend routine auditory monitoring as part of HIV care
Sebothoma (2020) [40]	Middle ear pathologies in adults within the mining industry: A systematic review	South Africa; mining settings	Adults working in mining	To review middle ear pathologies in miners	Middle ear issues are exacerbated by noise and comorbid conditions like HIV/TB	Highlights the need for middle ear assessments in noise-exposed populations
Khoza-Shangase (2010) [41]	HIV/AIDS and auditory function in adults: The need for intensified research in the developing world	South Africa; healthcare settings	HIV+ adults	To discuss gaps in research on HIV and auditory function in LMICs	Limited research highlights significant risks of auditory impairments in HIV	Calls for targeted studies in developing countries like South Africa
Assuiti et al. (2013) [42]	Hearing loss in people with HIV/AIDS and associated factors: An integrative review	Brazil; integrative review	HIV+ individuals	To identify the prevalence and associated factors of hearing loss in HIV+ individuals	High prevalence of hearing loss linked to disease progression, ART, and age	Emphasizes the importance of auditory health assessments in HIV care
Sagwa et al. (2015) [43]	Comparing amikacin and kanamycin-induced hearing loss in multidrug-resistant tuberculosis treatment	Namibia; retrospective cohort study	Patients with multidrug-resistant TB (MDR-TB) on aminoglycosides	To compare the ototoxic effects of amikacin and kanamycin	Both drugs caused significant hearing loss, with no notable differences between them	Highlights the need for safer TB treatments and regular hearing monitoring
Patel et al. (2001) [44]	Understanding barriers to preventive health actions for occupational noise-induced hearing loss	USA; survey study	Workers in noise-intensive industries	To identify barriers to preventive actions for NIHL	Lack of awareness and access to protective equipment contributes to NIHL	Advocates for improved education and resources for workers
Brits (2012) [45]	A description of the hearing profile in gold miners with tuberculosis	South Africa; mining settings	Gold miners with TB	To describe hearing profiles in TB-affected miners	TB treatments significantly worsened hearing thresholds	Recommends targeted auditory health interventions for miners with TB
Eisler (2003) [46]	Health risks of gold miners: A synoptic review	Global; mining settings	Gold miners worldwide	To review health risks, including hearing loss, among gold miners	Gold miners face compounded risks of NIHL and TB-related ototoxicity	Urges global standardization of health and safety protocols in mining
Kallail et al. (2008) [47]	Communication disorders in individuals with HIV/AIDS	USA; review study	HIV+ individuals	To explore the range of communication disorders in HIV+ patients	Auditory impairments are common, linked to both disease and treatment	Calls for comprehensive communication health services in HIV care
Harris et al. (2012) [48]	Aminoglycoside-induced hearing loss in HIV-positive and HIV-negative multidrug-resistant tuberculosis patients.	South Africa; clinical settings	Patients receiving TB treatment; HIV+ and HIV-	To compare ototoxicity in HIV+ and HIV- patients on TB treatment	High ototoxicity rates in both groups, with no significant difference by HIV status	Recommends routine hearing monitoring during TB treatment

## Data Availability

Data supporting the findings of this study are available within the paper.
